# Acute undifferentiated febrile illness in a traveler returning from Burkina Faso, West Africa to Puducherry, India- a case report

**DOI:** 10.1186/s12879-025-10869-8

**Published:** 2025-07-11

**Authors:** Srikanth Srirama, Krishan Kumar Sihag, Anand Kumar Chandrasekaran, Neethi Prasannan Latha, Waseema Arif, Balaji Sampath Kumar, Panneer Devaraju

**Affiliations:** 1 ICMR- Vector Control Research Centre, Medical Complex, Indira Nagar, Puducherry, 605006 Puducherry India; 2 Indira Gandhi Medical College and Research Institute, Puducherry, Puducherry India; 3 Present Address: Trivandrum Medical College, Trivandrum, Kerala India

**Keywords:** Imported infectious diseases, *Plasmodium falciparum*, Molecular diagnostic techniques, Travel medicine, Public health surveillance

## Abstract

**Background:**

Imported malaria remains a significant diagnostic challenge, particularly in regions approaching elimination. This case report is novel in its exploration of the complexities involved in diagnosing malaria imported from a high-endemic area to a low-endemic region, emphasizing the critical role of detailed travel history and molecular diagnostics in identifying the disease. The case underscores the potential public health implications of missing such diagnoses in regions where routine malaria testing is not performed due to low endemicity.

**Case presentation:**

A 25-year-old male presented to a tertiary care hospital in Puducherry, India, with symptoms of intermittent fever, headache, loss of appetite, and vomiting, which persisted over a week. Initial laboratory findings showed thrombocytopenia and leukopenia, with negative tests for dengue, chikungunya, and scrub typhus. Further investigation using molecular diagnostic techniques identified *Plasmodium falciparum*. The patient had recently returned from Burkina Faso, West Africa, where he had experienced similar episodes of fever, establishing the diagnosis of imported malaria.

**Conclusions:**

This case highlights the necessity of considering imported malaria in the differential diagnosis of febrile illnesses in India, a country nearing malaria elimination. It illustrates the importance of incorporating travel history into clinical evaluations and supports the use of molecular diagnostics to effectively diagnose malaria in settings where traditional diagnostic methods may overlook imported cases. The findings advocate for enhanced surveillance and diagnostic preparedness to manage imported cases of malaria, thereby supporting ongoing elimination efforts and preventing the re-establishment of local transmission.

**Clinical trial number:**

Not applicable.

## Introduction

Malaria is a public health concern for India and 84 other endemic countries across the world [1]. According to the World Malaria Report, 2023, India accounted for approximately 66% of the estimated number of malaria cases in the World Health Organization (WHO) South‒East Asia Region in 2022. As per the National Framework for Malaria Elimination, India is aligned with the global goal of malaria elimination by 2030, along with the prevention of re-establishment of transmission [2]. This would involve interrupting indigenous transmission of the disease across the country. There is also a need to improve surveillance systems to address the threat of imported malaria cases.

Here, we report a case of imported malaria in a traveler returning from Burkina Faso, West Africa, who presented with uncontrolled fever, raising concern about the threat of imported malaria. This case report aims to highlight the significance of a thorough travel history, a high index of suspicion for cases of fever from malaria-endemic countries or a history of travel from such endemic regions.

## The case

The patient, a 25-year-old male, presented with fever, loss of appetite, sweating, headache, dizziness, nausea and vomiting for one week at a government tertiary care medical college hospital in Puducherry on 22nd February 2023. The fever was intermittent and persistent but was reduced on antipyretic medications. He had no history of loose stools, joint pains, body aches or other comorbidities. He traveled to Wayanad in Kerala, India, one week before the onset of fever. There was no history of fever in his family.

Clinical and Diagnostic Assessment: On examination, the patient had a temperature of 37.4 °C with a pulse rate of 112/minute. The electrocardiogram was otherwise normal, and his blood pressure was 100/60 mmHg. His respiratory, cardiovascular and neurological examinations were normal. The abdomen was soft and nontender with no palpable organomegaly. Hematological analysis revealed thrombocytopenia (67000/mm^3^) and leukopenia (3100/mm^3^). The peripheral thick and thin blood smear examination was negative for malaria and microfilaria. By day 3 after admission (day 10 of fever onset) his temperature spiked to 38 °C; hemoglobin levels had fallen to 9.6 gm/dL, and his RBC count was 3.1 million/cumm (Table 1). The temperature was 38 °C on day 4 too, and reduced to 37.2–37 °C range for the remainder of his admission period. Liver and kidney function tests were normal, except for mild hypoalbuminemia. There was no evidence of hyperbilirubinemia. Blood and urine were sterile on culture. The tests for dengue, chikungunya and typhoid serology were negative (Table 2).

**Table 1:** Hematological profile of the patient since admission

Date	Days since fever started	Temperature (֯C)	Hb (gm/dL)	RBC Count (m/mm^3^)	PCV (%)	WBC Count (per mm^3^)	Platelets (per mm^3^)	Peripheral Smear for Malaria Parasites/ Microfilaria
**Laboratory Normal Reference Range**		**36.5–37.5**֯C	**12– 16 gm/dL**	**4.5–5.5 million/ mm** ^3^	**40–52%**	**4000–11,000/mm** ^3^	**150,000-400,000/ mm** ^3^	**Negative**
22-02-23 (Date of admission)	8	37.4	11.60_v_	3.9_v_	33.8_v_	3100_v_	67,000_v_	MP/MF- negative
23-02-23	9	37.4	10.50_v_	-	-	2900_v_	76,000_v_	Not done
24-02-23	10	38.2^	9.60_v_	3.1_v_	26.9_v_	3100_v_	76,000_v_	Not done
25-02-23	11	38.0^	10.80_v_	3.5_v_	30.0_v_	3600_v_	90,000_v_	Not done
26-02-23	12	37.2	11.00_v_	3.5_v_	30.3_v_	3700_v_	1,02,000_v_	Not done
27-02-23	13	37.0	10.80_v_	3.5_v_	29.5_v_	3600_v_	1,26,000_v_	Not done
28-02-23	14	37.0	11.00_v_	3.6_v_	30.8_v_	4100	1,56,000	MP/MF- negative
01-03-23 (Date of Discharge: 02-03-23)	15	37.0	11.40_v_	3.8_v_	32.0_v_	3900_v_	1,83,000	MP/MF- negative

_v_ Lower than normal; ^ Higher than normal; Hb- Hemoglobin in grams per decilitre; RBC- Red Blood Cell (Erythrocyte) count in millions per microlitre; PCV- Packed Cell Volume in percentage; WBC- White Blood Cell (Leukocyte) Count in number per microlitre; MP/MF- Malaria Parasite/ Microfilaria

**Table 2:** Other laboratory investigation reports of the patient

Sl.no	Investigation	Date	Result	Laboratory Normal Reference Range
1	Random Glucose	22-02-2023	80 mg/dL	60–100 mg/dL
2	Urea	22-02-2023	20 mg/dL	15–45 mg/dL
3	Creatinine	22-02-2023	1.2 mg/dL	0.5–1.4 mg/dL
4	Urine routine	22-02-2023	Normal, no albumin or sugars, 2–4 pus cells	No albumin or sugars, < 5 pus cells
5	Serum electrolytes	22-02-2023	Sodium- 137 mEq/L, Potassium- 3.5 mEq/L, Chloride- 109 mEq/L	136–145 mEq/L 3.5- 5 mEq/L 96–107 mEq/L
6	Hepatobiliary Profile	22-02-2023	Bilirubin Total- 0.9 mg/dL, Bilirubin Direct- 0.4 mg/dL, Protein total- 5.9 g/dL, Albumin- 3.7 g/dL, AST- 16 IU/L, ALT- 10 IU/L, ALP- 45 IU/L	0.2–1.3 mg/dL 0.1–0.4 mg/dL 6–8 g/dL 3.5–5 g/dL 5–46 IU/L 8–46 IU/L 15–306 IU/L
7	Serology	23-02-2023	Negative for Dengue NS1 Antigen	Negative
8	Serology	24-02-2023	Negative Dengue and Chikungunya IgM MAC ELISA	Negative
9	Widal test	24-02-2023	Negative [TO, TH, AH, BH < 1:80]	Negative
10	Bacterial Culture	25-02-2023	No growth in Blood and Urine	No Growth

AST- Aspartate Aminotransferase; ALT- Alanine Aminotransferase; ALP- Alkaline Phosphatase; NS1- Nonstructural Protein 1; IgM MAC ELISA- Immunoglobulin M Antibody Capture Enzyme-Linked Immunosorbent Assay; TO - Typhoid O Antigen; TH - Typhoid H Antigen; AH - Paratyphoid A H Antigen; BH - Paratyphoid B H Antigen

On day 5 after admission, 2 mL of venous blood in EDTA was sent to the ICMR-VCRC for the Vector-borne Diseases screening panel. DNA (DNeasy Blood and Tissue Kit) and RNA (RNeasy Kit) from patient blood samples were extracted using commercial kits (Qiagen, Germany). The sample tested negative for the arbovirus panel, which includes dengue, chikungunya, Japanese encephalitis and Zika viruses. It also tested negative for scrub typhus and spotted fever rickettsial pathogens by real-time PCR. The sample tested positive for malaria parasite by real-time PCR for *Plasmodium falciparum* [3]. Post PCR, blood smear microscopic examinations were repeated to look for malaria parasites but were negative again (Table 1), so the parasite density could not be determined.

Based on the PCR results, upon a detailed enquiry, the patient reported that he worked in Ouagadougou, Burkina Faso, West Africa, for one year and had returned three weeks before the onset of fever. During his stay in Burkina Faso, he had 3 episodes of fever lasting 5 days to one week each. His last bout of fever occurred in August 2022. As he had no further fever spikes, the patient was discharged on 2nd March after 9 days of admission.

**Fig. 1:**
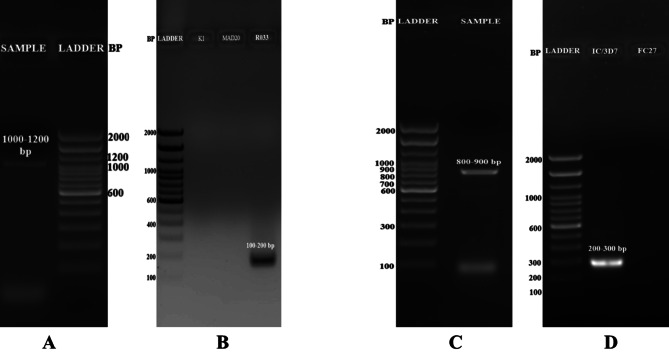
Agarose gel electrophoresis image of the PCR amplicons of (**A**- Lane 1: sample, Lane 2: 100 bp DNA ladder for reference) *msp1* gene corresponding to a band at 1000–1100 bp, (**B**- Lane 1: ladder, Lane 2- K1, Lane 3- MAD20, Lane 4- RO33) R033 allele of *msp1* corresponding to a band at 130–220 bp, (**C**- Lane 1: ladder, Lane 2: sample) *msp2* gene corresponding to a band at 800–900 bp and (**D**- Lane 1: ladder, Lane 2: 3D7, Lane 3: FC27) 3D7 allele of *msp2* corresponding to a band at 100–450 bp

**Fig. 2:**
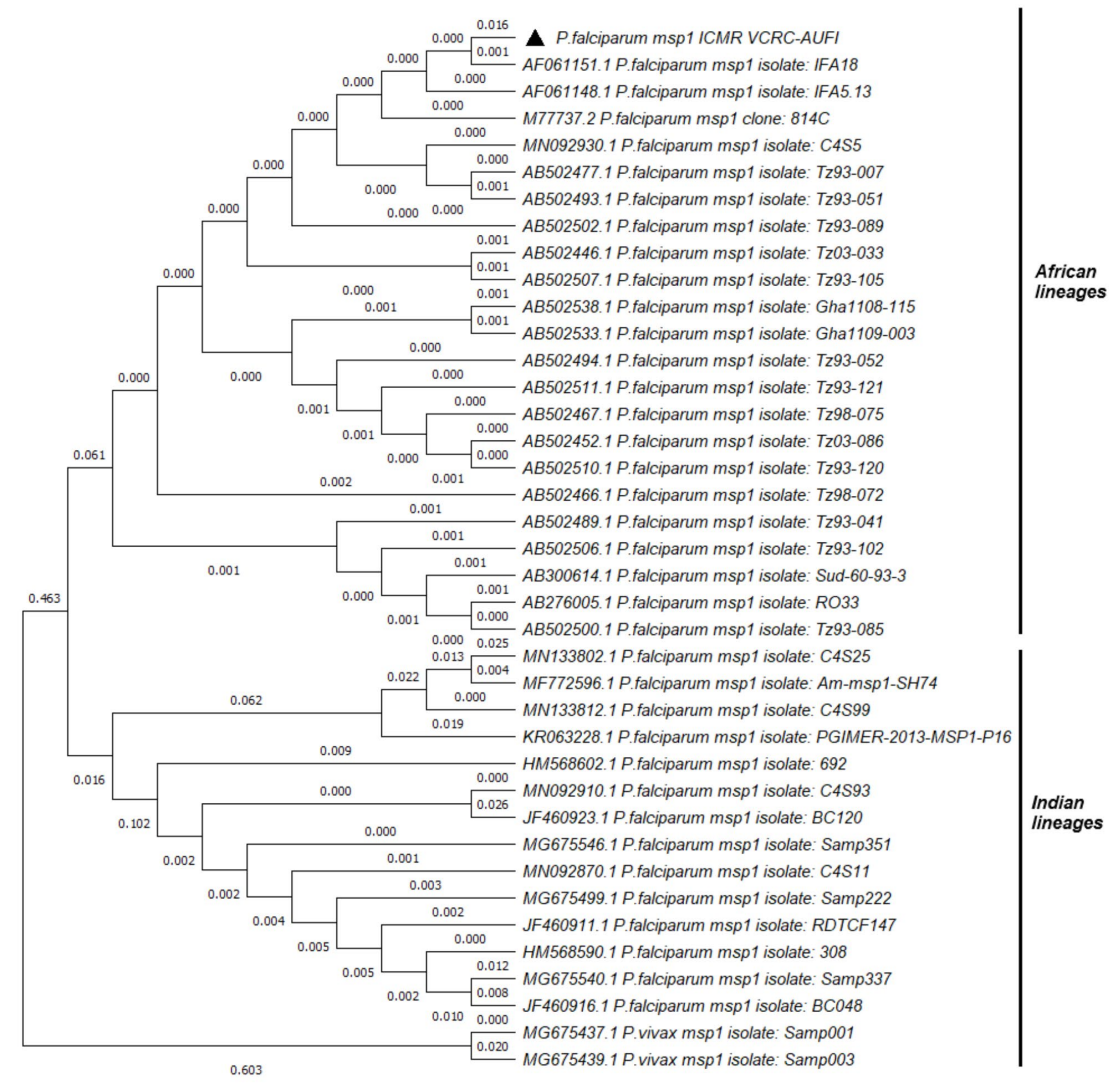
Phylogenetic distribution of *Plasmodium falciparum* based on the *msp1* nucleotide sequences. The phylogenetic tree compares the genetic sequences of different strains of *Plasmodium falciparum* based on the *msp1* gene variations reported in the global database and estimates how closely they are related to the strains from this study. It was constructed with 1000 bootstrap replicates by the maximum likelihood method using MEGA X. The *P. falciparum* detected in the patient in this study is represented by a solid triangle in front of the label

**Fig. 3:**
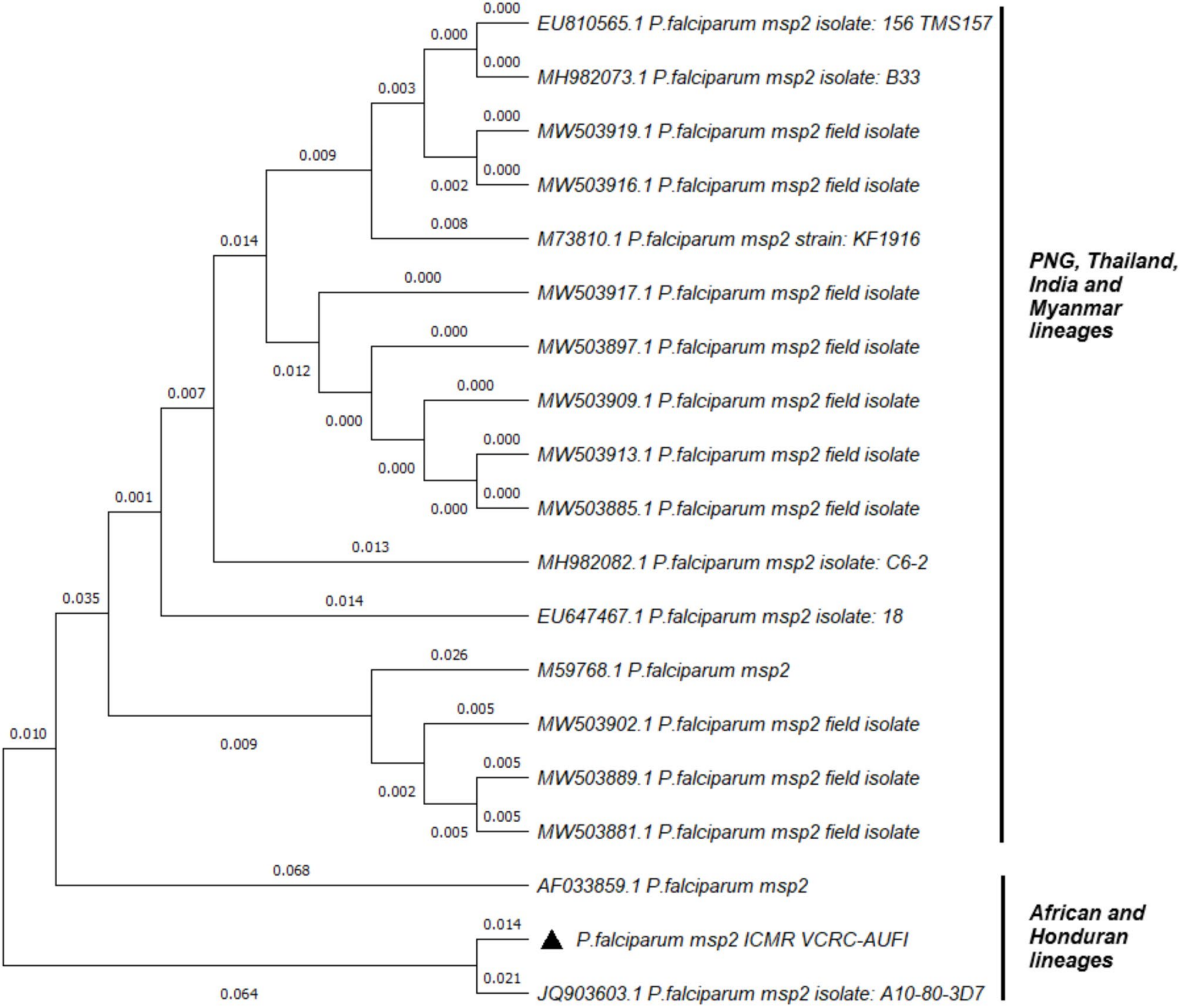
Phylogenetic distribution of *Plasmodium falciparum* based on the *msp2* nucleotide sequences. The phylogenetic tree compares the genetic sequences of different strains of *Plasmodium falciparum* based on the *msp2* gene variations reported in the global database and estimates how closely they are related to the strains from this study. It was constructed with 1000 bootstrap replicates by the maximum likelihood method using MEGA X. The *P. falciparum* detected in the patient in this study is represented by a solid triangle in front of the label

The clonal composition of the tested *Plasmodium falciparum* strains was analyzed by PCR using allele-specific primers to amplify conserved block 2 for the *msp1* gene and block 3 for the *msp2* gene (Fig. 1A and C) [4]. The parasite had R033 and 3D7 alleles in the *msp1* and *msp2* genes, respectively. (Fig. 1B and D). A phylogenetic tree was constructed using the maximum likelihood algorithm using the Kimura-2 parameter with 1000 bootstrap values on MEGA X software using the nucleotide sequences of the *msp1* and *msp2* genes. (Figs. 2 and 3).

## Discussion

A vital socioeconomic factor overlooked in malaria control is the large migration of Indians to malaria-endemic countries. Approximately 13.45 million Indians are overseas expatriate workers, students, business people, etc [5]. These individuals are vulnerable to active malarial infection in other endemic countries.

Despite initial differential diagnoses of dengue, scrub typhus and typhoid based on clinical presentation and seasonal trends, malaria was ultimately detected through molecular methods in this patient. The molecular positivity for malaria explained the symptoms and the haematological picture. Only upon subsequent questioning, the underlying history of travel with fever and mosquito bite exposure in Burkina Faso was elucidated.

India faces a dual challenge in its fight against malaria, grappling with both indigenous and imported cases, which hinders its journey toward malaria elimination. With extensive population movements across borders, the risk of reintroduction of the parasite through these cases is notable, as seen in instances across countries such as Spain, Italy, Germany, and France [6–9]. The first local transmission of malaria in the USA in 20 years is believed to be due to imported cases [10]. Saudi Arabia and Sri Lanka have eliminated or nearly eliminated malaria transmission locally and have surveillance plans for pilgrims, migrants and foreign workers [11, 12]. Sri Lanka also faced the risk of malaria in military personnel returning from endemic regions [13]. China has reported over 1053 imported malaria cases in Shandong province between 2013 and 2017 [14]. UK has reported about 1400 imported cases yearly between 2012 and 2021, with 1012 cases in 2021 [15]. Australia has reported 3204 imported cases between 2012 and 2022, with most cases from sub-Saharan Africa [16]. A meta-analysis of over 50,000 cases from non-endemic countries has revealed that over 92% of 24,941 imported cases originated from West Africa (56%), India (20%), East Africa (13%) and Papua New Guinea (3%) during 2005- 2015 [17]. While most of these reported cases belonged to immigrants and travelers, the emigrated population returning with an infection is possibly overlooked.

Puducherry reported 15, 5 and 0 cases of malaria in 2020, 2021 and 2022, respectively, all of which were *P. vivax* [18]. Despite controlling local malaria, Puducherry faces a significant risk of potential upsurges from migrating and imported cases and traveling from other endemic regions. Furthermore, transmission is favored by the presence of the urban malaria vector *Anopheles stephensi* [19]. As Puducherry is currently a low-endemic setting, rapid diagnostic tests (RDTs) and microscopy are not routinely performed for febrile illness patients. Microscopy may fail to detect malaria infections accurately due to its dependence on smear quality, staining, fixation, and the expertise of the technicians, especially in cases with low parasite load in the blood. Furthermore, microscopy may underestimate malaria cases in regions with an overall low malaria load [20]. In Puducherry, there is an annual malaria microscopy training, but this is usually for the staff of the national program and public health laboratories. The staff of government colleges can also participate in this training after obtaining due permission, however it is not mandatory. Coupled with lower malaria cases, the staff training could have contributed to the negative smear examination report in this case. Proactive surveillance of travelers is critical to reduce transmission risks from globally mobile disease reservoirs and provide optimal individual and public health outcomes. Hence, RT-PCR-based molecular diagnostic methods may be used to aid in rapid screening of parasites and drug resistance. The RT-PCR-equipped public health laboratories network setup for COVID-19 diagnosis across the country could be strengthened to achieve this goal with a shorter turnaround time. Microscopy coupled with PCR in low-endemic settings, as seen in Spain, would be beneficial if adopted in India [6]. 

The two merozoite surface proteins *msp1* and *msp2* of the *P. falciparum* parasite are coded by single locus genes. Alleles, variants of nucleotide sequence caused by polymorphism, reported in these genes are widely used as markers for genotyping the different strains of the parasite. Three distinct allelic families in block 2 of *msp1* (K1, MAD20, RO33) and two distinct families in block 3 of *msp2* (FC27, 3D7) have been recognised and PCR to amplify them have been developed. Our study adopted the protocol described by Some et al., 2018 to amplify these polymorphic genes [4]. The presence of a single allele in *msp1* (RO33) and *msp2* (IC/3D7) genes indicates infection with a single strain of the parasite. Furthermore, by phylogenetic analysis, the allele clustered with the African isolates, confirming that the infection was not indigenous but rather acquired in Burkina Faso, West Africa (Figs. 2 and 3**).** Studies from Burkina Faso have reported the presence of the RO33 (36%) and IC/3D7 (88.2%) allelic families in the *msp1* and *msp2* genes [4]. Furthermore, Soulama et al.. (2009) reported that the 3D7 allelic family was commonly present in symptomatic malaria patients in urban settings, which is consistent with the history of patients staying at Ouagadougou [21]. While the significance of RO33 and 3D7 alleles isn’t fully explored in terms of resistance patterns, they are linked to specific clinical outcomes like severe malaria and liver function abnormalities in the presence of multiple alleles, in a study from Indonesia [22]. An older study, from Papua New Guinea, suggests that multiple infections may offer some level of protective immunity, potentially by eliciting a broader immune response [23]. The protective role of these specific alleles, particularly *3D7* and *RO33*, may be linked to immune responses targeting polymorphic epitopes in the merozoite surface proteins, though this requires further investigation. The findings support the idea that concurrent infections may enhance immune protection, reducing the likelihood of severe malaria outcomes.

This case underlines the importance of the proper acquisition of a longer travel history as a pivotal component to guide sound differential diagnosis, on admission. If travel history is neglected, exotic pathogens with different drug resistance profiles might spread to the indigenous population. A detailed history and the maintenance of accessible medical records for travelers can enable prompt diagnosis and treatment of imported cases. Clinicians should be sensitized to suspect malaria in the context of a positive travel history to endemic regions. Surveillance of mobile populations, preventive measures such as pre-travel counseling with prophylaxis, and molecular diagnostics to identify parasitic drug resistance are vital for malaria elimination.

While RDTs are not used in non-endemic areas by the national vector-borne diseases control program, there should be a rational plan to proactively screen symptomatic patients with a history of travel to endemic areas, using bivalent RDT, microscopy and molecular methods. Regular training of all relevant healthcare staff, in primary, secondary and tertiary care settings on good-quality microscopy is required to sustain the elimination efforts. Rapid detection of imported malaria is crucial for preventing potential malaria outbreaks in areas with abundant vectors that could undermine elimination goals. The diagnosis of imported malaria across varying endemicity settings holds significant public health implications. In non-endemic or low-endemic regions, it raises concerns of reintroducing local transmission, especially if competent vectors exist, and poses diagnostic challenges due to limited healthcare experience and skills with microscopy, potentially causing delays in treatment and management [20]. In endemic regions, imported cases can complicate control efforts, especially if they involve drug-resistant strains or non-local parasite species, necessitating adjustments in treatment protocols and resource allocation. Surveillance and vector control strategies must focus on ensuring access to diagnosis and treatment, particularly in labor camps or migrant shelters as laid out in the National Strategic Plan for Malaria Elimination in India [24]. Care should also be taken to include the local migrants who have returned from other endemic settings. While the National Strategic Plan [24] does suggest molecular tests for confirmation and operational research when needed, it is also important to include molecular tests for malaria diagnosis in diagnostic protocols for malaria surveillance, as this case illustrates. Integrated vector management in high-risk areas, including indoor residual spraying (IRS) and long-lasting insecticidal nets (LLINs), is crucial, as is increasing awareness through social and behavior change communication (SBCC) initiatives tailored to the migrant population. Collaboration with labor, housing, and urban development sectors, along with interstate cross-border consultation, is essential for monitoring and controlling the spread of malaria, contributing to sustained malaria elimination efforts [25]. 

Across all settings, managing imported malaria requires robust surveillance systems, rapid diagnostics, vector control, community education, and international collaboration to share data on parasite characteristics and resistance patterns. Effective intervention and preparedness in both low and high-endemic regions are essential to reducing transmission risk, safeguarding health security, and preventing outbreaks.

## Data Availability

The datasets of the sequences and images used and/or analysed during the current study are available from the corresponding author upon reasonable request.

## Abbreviations

ICMR-VCRC: Indian council of medical research-vector control research centre
PCR: Polymerase chain reaction
DNA: Deoxyribonucleic acid
RNA: Ribonucleic acid
RDTs: Rapid diagnostic tests
RT: PCR-reverse transcription polymerase chain reaction
WHO: World health organization
msp1: Merozoite surface protein 1
msp2: Merozoite surface protein 2
IHEC: Institutional human ethics committee
IRS: Indoor residual spray
LLIN: Long-lasting insecticidal net
SBCC: Social and behaviour change communication
NS1: Non-structural protein 1
IgM: Immunoglobulin M
ELISA: Enzyme-linked immunosorbent assay
Hb: Hemoglobin
RBC: Red blood cell
PCV: Packed cell volume
WBC: White blood cell
MP: Malaria parasite
MF: Microfilaria
ALP: Alkaline phosphatase
AST: Aspartate aminotransferase
ALT: Alanine aminotransferase
